# A lncRNA-disease association prediction tool development based on bridge heterogeneous information network via graph representation learning for family medicine and primary care

**DOI:** 10.3389/fgene.2023.1084482

**Published:** 2023-05-18

**Authors:** Ping Zhang, Weihan Zhang, Weicheng Sun, Li Li, Jinsheng Xu, Lei Wang, Leon Wong

**Affiliations:** ^1^ Hubei Key Laboratory of Agricultural Bioinformatics, College of Informatics, Huazhong Agricultural University, Wuhan, China; ^2^ Guangxi Key Lab of Human-Machine Interaction and Intelligent Decision, Guangxi Academy of Sciences, Nanning, China; ^3^ Institute of Machine Learning and Systems Biology, School of Electronics and Information Engineering, Tongji University, Shanghai, China

**Keywords:** lncRNA-disease associations, disease, graph representation learning, bridge heterogeneous information, SDNE, family medicine and primary care

## Abstract

Identification of long non-coding RNAs (lncRNAs) associated with common diseases is crucial for patient self-diagnosis and monitoring of health conditions using artificial intelligence (AI) technology at home. LncRNAs have gained significant attention due to their crucial roles in the pathogenesis of complex human diseases and identifying their associations with diseases can aid in developing diagnostic biomarkers at the molecular level. Computational methods for predicting lncRNA-disease associations (LDAs) have become necessary due to the time-consuming and labor-intensive nature of wet biological experiments in hospitals, enabling patients to access LDAs through their AI terminal devices at any time. Here, we have developed a predictive tool, LDAGRL, for identifying potential LDAs using a bridge heterogeneous information network (BHnet) constructed via Structural Deep Network Embedding (SDNE). The BHnet consists of three types of molecules as bridge nodes to implicitly link the lncRNA with disease nodes and the SDNE is used to learn high-quality node representations and make LDA predictions in a unified graph space. To assess the feasibility and performance of LDAGRL, extensive experiments, including 5-fold cross-validation, comparison with state-of-the-art methods, comparison on different classifiers and comparison of different node feature combinations, were conducted, and the results showed that LDAGRL achieved satisfactory prediction performance, indicating its potential as an effective LDAs prediction tool for family medicine and primary care.

## Introduction

Autonomously understanding illness or physical condition for body fluid biomarker samplers is significant after getting the medical report for family medicine and primary care. It is self-diagnostic for patients to know their health conditions through artificial intelligence (AI) at home. The fluid biomarkers, such as non-coding RNA molecules, are that we often need to be tested in the course of disease prevention and treatment. Among various non-coding RNA molecules, one of the most essential and unique non-coding RNA molecules with longer than 200 nucleotides, long non-coding RNAs (lncRNAs), was initially thought to be transcriptional noise. Recently, with remarkable technologies such as developed sequencing newly, more and more lncRNAs have been identified, and their functions associated with multiple diseases have received much attention (Yanofsky, 2007; Core et al., 2008; Lv et al., 2014). For instance, for some cancers such as lung cancer, bladder cancer, breast cancer, and colorectal cancer, lncRNA-UCA1 is expressed at high levels during diagnosis and treatment (Wang et al., 2015). Besides, the lncRNA PCA3, as a potential cancer diagnostic biomarker, is also a well-known example. Researchers have found that PCA3 expression levels significantly increased in prostate tumors compared with normal tissues (Spizzo et al., 2012; van Poppel et al., 2012). Hence, it can help to understand the occurrence of diseases and the development process and further facilitate the diagnosis, treatment, and prevention of human diseases by detecting potential LDAs. However, wet biological experiments have inherent weaknesses: time consumption, low efficiency, and high cost. It is imperative to build accurate and effective computational models for predicting potential lncRNAs related to diseases.

Recently, computational models have been proposed and have become powerful tools for predicting LDAs. Given the implementation strategy, most existing LDAs prediction approaches can predominantly be summarized into three categories: The first category is based on machine learning methods. They used known disease-related lncRNAs to infer new associations by an efficient feature engineering algorithm. For example, according to the initial probability vector of known LDAs, an improved IRWRLDA model was proposed by Chen et al., where they combined disease semantic similarity with lncRNA expression similarity using the Random Walk algorithm to predict unknown LDAs (Chen et al., 2016). Yu et al. proposed a computational model called NBCLDA to detect potential LDAs via the naive Bayesian classifier (Yu et al., 2018). Chen et al. used random projection combined with a finite impulse response filter to predict self-interacting proteins (Chen et al., 2018). Ou-Yang et al. employed a two-side sparse self-representation algorithm to estimate representations of lncRNA and disease for LDAs (Ou-Yang et al., 2019). Han et al. proposed a gene selection method called BPSO via binary particle swarm optimization and prior information (Han et al., 2015). Zheng et al. adopt consensus-independent component analysis for Gene expression data classification (Zheng et al., 2008). Besides, some prediction models associated with LDAs prediction, such as protein-protein interactions prediction (Huang and Zheng, 2006; Zheng et al., 2008; Xia et al., 2010b; 2010a; Shi et al., 2010; You et al., 2010; Zhu et al., 2013; Huang et al., 2014), took advantage of machine learning methods to predict protein-protein interactions based on multi-biometric features. The second category is based on matrix decomposition, in which they predict associations between molecules through decomposing and recovering low-rank matrix. For example, Lu et al. utilized an inductive matrix completion method to predict LDAs (Lu et al., 2018). Zheng et al. applied the penalized decomposition to gene expression data to extract meta-samples for clustering and identify the samples with complex classes (Zheng et al., 2011). As we know, the third category can be regarded as network-based methods. To achieve satisfactory performances, these network-based methods such as (Yang et al., 2011), (Sun et al., 2014), and (Zhou et al., 2015) integrated relationships networks, including known lncRNA-disease associations, disease similarity networks, and lncRNA similarity networks to build a heterogeneous network and then propagation algorithm is used for node embedding learning. With the development of a bipartite/tripartite graph with similarity networks as a heterogeneous network-based approach, Ding et al. propose a TPGLDA model by constructing a lncRNA-disease-gene tripartite graph (Ding et al., 2018). Based on the tripartite graph, Mori et al. incorporated biological sequence information into a disease-target-ncRNA tripartite network to predict ncRNA-disease associations (Mori et al., 2018). In addition, Ping et al. proposed a model to infer potential LDAs by constructing a bipartite network that follows the principle of a power-law distribution (Ping et al., 2018). In (Sumathipala et al., 2019), a complex multi-level network called LION in which protein-disease associations, protein-protein interactions, and lncRNA-protein interactions are jointly constructed, and the Random Walk algorithm is also utilized to learn node embedding. Regarding bio-network, Deng et al. predicted hub genes associated with cervical cancer via gene co-expression networks (Deng et al., 2015). Yuan et al. used bi-weight mid-correlation to measure the correlation between factors and then utilized nonconvex penalty-based sparse regression to infer the gene regulatory network (Yuan et al., 2018). Zhu et al. employed local similarity-preserving embedding to identify spurious interactions in the protein-protein interaction networks (Zhu et al., 2015).

Although predictive results of network-based models adopted bipartite or tripartite graphs were helpful to some extent, from another perspective, this also indicates that the relevance between lncRNA and diseases is a complex biological process in which many factors are closely involved. Besides, it is worth pointing out that the occurrence and development of complex diseases are also complex biochemical reactions involving many biomolecules. Thus, it is meaningful and essential to investigate the association role of multiple relevant molecules between lncRNA and disease. By integrating multiple molecule associations, Guo et al. proposed a novel molecular associations network model (Guo et al., 2019). Based on the DeepWalk algorithm, Chen et al. also proposed a prediction model for drug–target interactions from a multi-molecular network (Chen et al., 2020). These methods demonstrate that multiple molecule networks might be a powerful prediction method. It is worth mentioning that which molecules could be adopted is also challenging. Different research objects will have different opinions on this point.

Due to the in-depth research in LDAs prediction, the current network-based approaches are now regarded as a powerful alternative. In this paper, inspired by the study of graph deep learning, for the LDAs prediction issue, we try to pick lncRNA, miRNA, drug, protein, and disease to construct a bridge heterogeneous information network (BHnet) based on the competing endogenous RNAs (ceRNA) hypothesis, which is biologically meaningful and rich in regulatory relationship with lncRNA. So, a novel model termed LDAGRL was proposed to predict potential LDAs by proposed BHnet including nodes (lncRNA, miRNA, drug, protein, and disease) and edges (the relationships among nodes). For exploration, LDAGRL aimed to take advantage of the multi-molecular network to verify that it can achieve satisfactory predictive performance. To better estimate the prediction performance of LDAGRL, comprehensive experiments, including 5-fold cross-validation (5-CV), comprehensive comparison with baselines, comparison on different classifiers and comparison of different node feature combinations, have been implemented. As a result, the 5-CV results show that our method obtains a satisfactory prediction performance, demonstrating that LDAGRL has promised performance in potential LDAs prediction.

## Materials and methods

### Datasets

**FIGURE 1:**
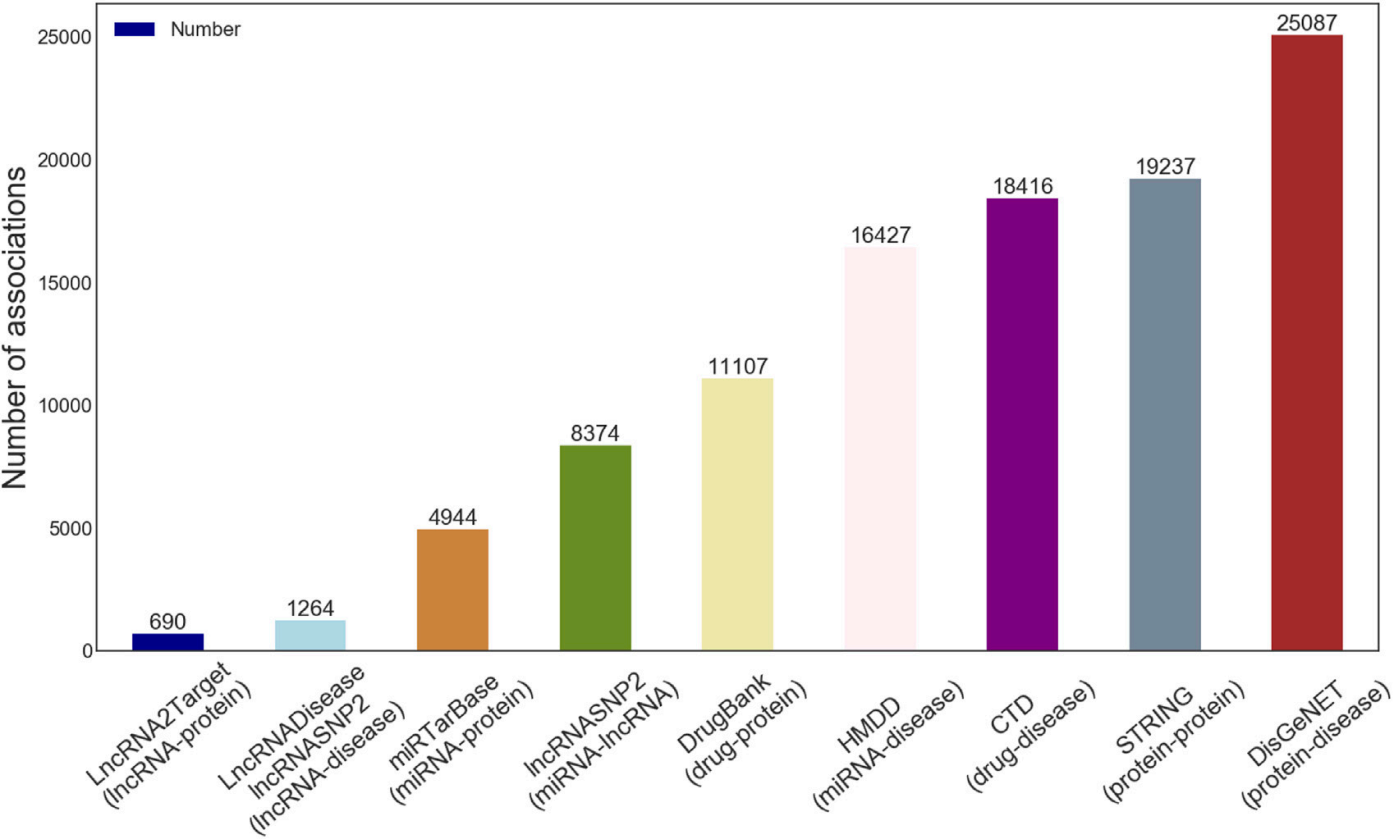
The details of nine kinds of associations in the LDAGRL.

According to the actual situation, we first download eight known kinds of associations from multiple databases. Then a set of data pre-processing operators, including identifier unification, de-redundancy, and deletion of the irrelevant items, are implemented. Besides, we gathered known experimentally supported LDAs data from the lncRNASNP2 and the LncRNADisease database, and we thus obtained 345 different lncRNAs and 295 different diseases (i.e., 1264 independent lncRNA-disease association pairs as positive samples). The details of the final LDAGRL objects data are shown in Table 1 and Figure 1.

**TABLE 1:** The databases of nine kinds of associations in the LDAGRL.

Relationship type	Database
lncRNA-disease	LncRNADisease (Chen et al., 2012) lncRNASNP2 (Miao et al., 2018)
miRNA-lncRNA	lncRNASNP2 (Miao et al., 2018)
lncRNA-protein	LncRNA2Target (Cheng et al., 2019)
miRNA-disease	HMDD (Huang et al., 2019)
Protein-disease	DisGeNET (Piñero et al., 2016)
Drug-disease	CTD (Davis et al., 2013)
miRNA-protein	miRTarBase (Chou et al., 2018)
Drug-protein	DrugBank (Wishart et al., 2018)
protein-protein	STRING (Szklarczyk et al., 2016)

### Experiment evaluation

The prediction performance of LDAGRL was evaluated mainly using the area under the receiver operating characteristic curve (AUC). Relevant evaluation metrics include Accuracy (Acc.), Precision (Prec.), Sensitivity (Sen.) or Recall, Specificity (Spec.) F1-Score and MCC (Matthews correlation coefficient) and their definitions as follows:

$$Accuracy\;\left(Acc.\right)=\frac{TP+TN}{TP+TN+FP+FN}$$ (1)

$$Precision\left(Prec.\right)=\frac{TP}{TP+FP}$$ (2)

$$Sensitivity\left(Sen.\right)=Recall=\frac{TP}{TP+FN}$$ (3)

$$Specificity\;\left(Spec.\right)=1-\frac{TP}{FP+TN}$$ (4)

$$F1-Score=\frac{2\times Precision\times Recall}{Precision+Recall}$$ (5)

$$Matthews\;correlation\;coefficient\;\left(MCC\right)=\frac{TP\times TN-FP\times FN}{\sqrt{\left(TP+FP\right)\left(TP+FN\right)\left(TN+FP\right)\left(TN+FN\right)}}$$ (6)

where *TP*, *FP*, *TN*, and *FN* respectively represent the number of true positives, false positives, true negatives, and false negatives.

We adopted eight out of nine kinds of associations to construct BHnet. Then, the bridge feature of the node can be obtained by graph embedding algorithm on the BHnet. Note that LDAs are not included in the BHnet such that we can explore the potential relationship possibility between the lncRNA nodes and the disease nodes in the case of no prior edges (LDAs) in the BHnet for LDAGR. In other words, this article sets out to explore the association possibilities between lncRNAs and diseases only relying on their bridge nodes. Hence, we used LDAs as a training set and test set to conduct 5-CV, while the bridge feature (learned from eight types of associations) was the node/edge feature.

### LDAGRL overview

**FIGURE 2:**
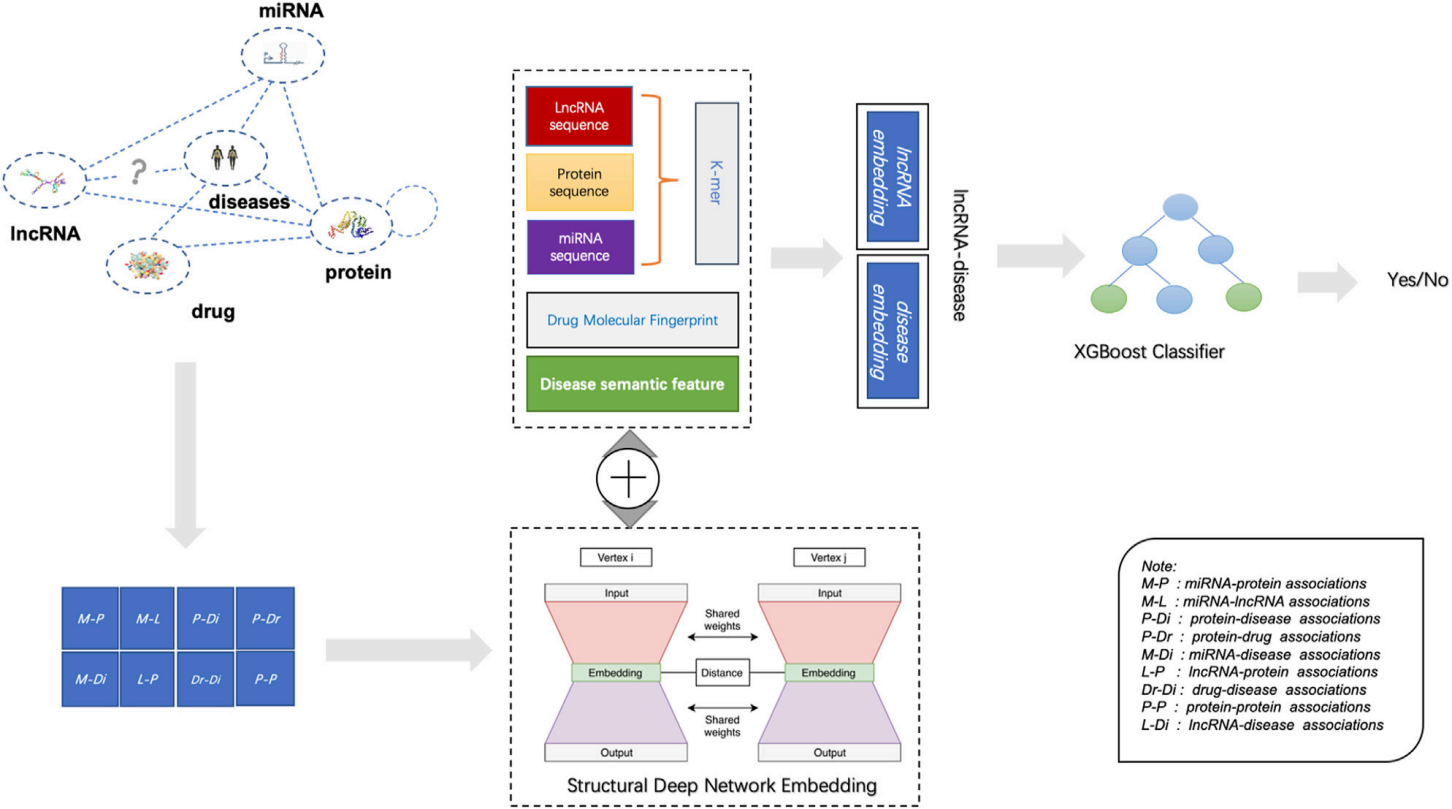
Flowchart of LDAGRL for predicting LDAs.

According to available datasets, the proposed BHnet based on biomolecules can be composed of nodes and edges. For nodes, there are five kinds of molecular such as ncRNA (miRNA, lncRNA), protein (target), drug, and disease. For edges, it consists of eight associations except known lncRNA-disease associations. Since the unknown lncRNA disease association would have been predicted, eight associations were embedded in BHnet. As shown in Figure 2, LDAGRL consists of three parts. First, we construct a BHnet by integrating biomolecule data, including five types of molecules. Second, we leveraged SDNE to learn node representations (node embedding) as dense feature vectors for LDA pairs. Third, we used a supervised machine learning-based XGBoost classifier to predict unknown LDAs.

Specifically, in the LDAGRL, by integrating multiple molecules information, we leveraged multiple relationships (associations) to construct a BHnet for lncRNAs and diseases, including eight kinds of associations (i.e., miRNA-lncRNA, miRNA-disease, lncRNA-protein, protein-disease, drug-disease, miRNA-protein, drug-protein, protein-protein). Then, based on the graph embedding framework, we employed SDNE to learn the embedding vector of nodes. Finally, we adopted the embedding vector combined with the positive and negative samples constructed by LDAs to train the XGBoost classifier, aiming to predict potential links.

### LncRNA and protein sequence representation

As shown in Figure 1, we downloaded the sequences information of lncRNA, miRNA, and protein from miRbase (Kozomara et al., 2019), NONCODE (Fang et al., 2018), and STRING (Szklarczyk et al., 2016) database respectively. Similar to the previous methods, we utilize a 64-dimensional vector to encode ncRNA (i.e., lncRNA and miRNA) sequences, where each attribute in nodes represents the normalized frequency of the k-mer for corresponding sequences. Based on the polarity of the side chain, we first divide 20 amino acids into four types and then represent each protein sequence by k-mer to form a 64-dimensional vector following the method proposed by Shen et al. (Shen et al., 2007).

### Drug molecular fingerprint representation

In LDAGRL, the smiles of drugs are downloaded from the DrugBank database (Wishart et al., 2018), which combines detailed drug data with comprehensive drug target information. By RDKit (Open-Source Cheminformatics Software) API, we transform the smiles of drugs into corresponding Morgan fingerprints to get drug molecular fingerprint representation.

### Disease semantic feature

The MeSH (Medical Subject Headings) is a comprehensive searchable control vocabulary primarily employed for indexing journal articles and books in the life sciences (Wang et al., 2010). In MeSH, related disease annotation terms can be represented by a Directed Acyclic Graph (DAG) structure that can be expressed as *DAG* = (*D*, *N*(*D*), *E*(*D*)). The *D*_*d*_(*t*) of a disease *t* in a DAG to the semantics of disease *D* is defined as follows:

$$\left\{\begin{array}{c} D_{d}\left(D\right)=1\\ D_{d}\left(t\right)=\max\left\{0.5*D_{d}\left(t^{\prime }\right)|t^{\prime }\epsilon \;children\;of\;t\right\}\;if\;t\neq d \end{array}\right.$$ (7)

where for a given disease *D, N*(*D*) denotes *D* itself together with all its ancestor nodes, while *E*(*D*) denotes all relationships connecting between nodes in the *DAG*(*D*). So, the semantic feature score between two diseases, where the *i* and *j*, can then be calculated by:

$$S\left(i,j\right)=\frac{\sum_{t\in T\left(i\right)\cap T\left(j\right)}\left(D_{i}\left(t\right)+D_{j}\left(t\right)\right)}{\sum_{t\in T\left(i\right)}D_{i}\left(t\right)+\sum_{t\in T\left(j\right)}D_{j}\left(t\right)}$$ (8)

### Structural Deep Network Embedding

Numerical studies substantiate the effectiveness and superior abilities of the proposed Structural Deep Network Embedding (SDNE)(Wang et al., 2016), which is a semi-supervised deep model to perform network embedding. It can preserve the highly-nonlinear local-global network structure well and is robust to sparse networks, with its advantages mainly focusing on the following crucial two points: A deep architecture: To capture the highly nonlinear network structure, it is composed of multiple nonlinear mapping functions to map the input data to a highly nonlinear latent space to capture the network structure; A semi-supervised model: To address the structure-preserving and sparsity problems, it exploits both the second-order and first-order proximity. Meanwhile, it designed the unsupervised component to preserve the first- and second-order proximity to refining the representations in the latent space.

Here, unsupervised components preserve global network structures by second-order proximity. As an unsupervised model, Autoencoder consists of two parts, i.e., the encoder and decoder. The Autoencoder aims to minimize the output and input reconstruction error. For given the input *x* ^*i*^, for each layer, the hidden representations are shown as follows:

$${y_{i}}^{\left(1\right)}=\sigma \left(W^{\left(1\right)}x^{i}+b^{\left(1\right)}\right),\;k=1$$ (9)

$${y_{i}}^{\left(k\right)}=\sigma \left(W^{\left(k\right)}{y_{i}}^{\left(k-1\right)}+b^{\left(k\right)}\right),\;k=2,\ldots ,K$$ (10)

After obtaining ${y_{i}}^{k}$ , we can obtain the output ${\hat{x}}_{i}$ by reversing the calculation process of the encoder. The objective function is shown as follows:

$$O=\sum_{n=1}^{\infty }{∥{\hat{x}}_{i}-x_{i}∥}_{2}^{2}$$ (11)

Considering the penalty or regularization, more penalty to the reconstruction error of the non-zero elements than that of zero elements in the adjacency matrix. The revised objective function is shown as follows:

$$O_{2nd}=\sum_{n=1}^{\infty }{∥\left({\hat{x}}_{i}-x_{i}\right)⊙b_{i}∥}_{2}^{2}$$ (12)

$$={∥\left(\hat{X}-X\right)⊙\beta ∥}_{F}^{2}$$

where⊙means the Hadamard product, *b*_*i*_ *=* ${\left\{b_{i.j}\right\}}_{j=1}^{n}.if\;s_{i,j}=0,b_{i,j}=1,else\;b_{i,j}=\beta >1$

It is essential to preserve the local structure. Therefore, the supervised component is designed to exploit the first-order proximity. The objective function for this goal is defined as follows:

$$O_{1st}=\sum_{i,j=1}^{n}s_{i,j}{∥{y_{i}}^{\left(k\right)}-{y_{j}}^{\left(k\right)}∥}_{2}^{2}$$ (13)

$$=\sum_{i,j=1}^{n}s_{i,j}{∥y_{i}-y_{j}∥}_{2}^{2}$$

To preserve the first-order and second-order proximity simultaneously, a semi-supervised model was proposed, which combines Eq. 12 and Eq. 13, and joint minimizes the following objective function:

$$O_{mix}=O_{2nd}+{\alpha O}_{1st}+{\nu L}_{reg}$$ (14)

$$={∥\left(\hat{X}-X\right)⊙B∥}_{F}^{2}+\alpha \sum_{i,j=1}^{n}s_{i,j}{∥y_{i}-y_{j}∥}_{2}^{2}{+\nu L}_{reg}$$

where $L_{reg}$ is an L 2-norm regularizer term to prevent overfitting, which is defined as follows:

$$L_{reg}=\frac{1}{2}\sum_{k=1}^{K}\left({∥W^{\left(k\right)}∥}_{F}^{2}{+∥{\hat{W}}^{\left(k\right)}∥}_{F}^{2}\right)$$ (15)

## Results and discussion

### Cross-validation experiment

In this section, to demonstrate the prediction performance of our novel method, the bridge molecular, including lncRNA, miRNA, drug, and protein, are integrated to obtain lncRNA-disease link embedding. Thus, we utilize SDNE to train the proposed BHnet and to get a dense representation of the lncRNA and the disease vector.

**FIGURE 3:**
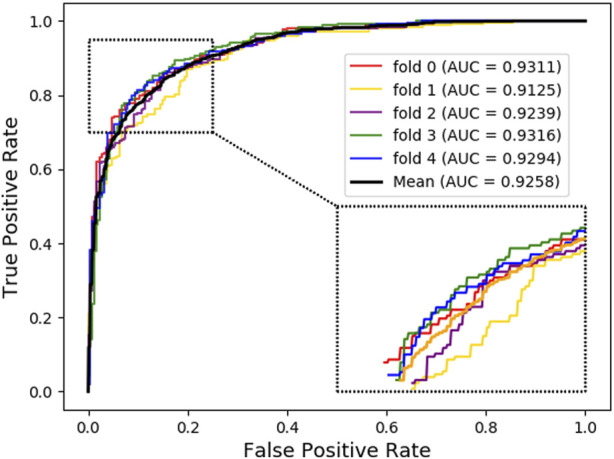
AUC of LDAGRL using SDNE network representation method via XGBoost classifier.

For further investigation, we choose XGBoost as a classifier algorithm to verify the classification performance of LDAGRL. Moreover, the AUC scores are used to evaluate the predictive performance of our method. As seen from Table 2 and Figure 3, as illustrated in the method section, LDAGRL effectively predicts potential lncRNAs related to diseases. Specifically, it can be easily found that LDAGRL achieved a reliable AUC of 0.9258, which is the expected AUC we required. We can see that in most previous methods, the characteristics of the research objects themselves were considered to detect unknown relationships in these methods.

**TABLE 2:** 5-CV results of LDAGRL.

Fold	Evaluation metrics				
	Acc	Sen	Spec	Prec	MCC
0	0.8458	0.8458	0.8458	0.8458	0.6917
1	0.8162	0.7826	0.8498	0.8390	0.6338
2	0.8360	0.8182	0.8538	0.8484	0.6724
3	0.8557	0.8419	0.8696	0.8659	0.7117
4	0.8492	0.8413	0.8571	0.8548	0.6985
Average	0.8406	0.8260	0.8552	0.8508	0.6816

Nevertheless, there are many other indirect associations, such as the other associations in LDAGRL. If these previous methods integrate these different indirect associations, the predictive performance can be improved significantly. Our method is better than previous methods (such as bipartite graph) that only focus on single or isolated objects to some extent. As can be seen, LDAGRL works more effectively in predicting potential LDAs when adopting multiple bridge relationships-based methods.

### Comparison with state-of-the-art methods

Then, we compared our model with existing state-of-the-art methods, i.e., GCNLDA (Xuan et al., 2019), GCRFLDA (Fan et al., 2022) and gGATLDA (Wang and Zhong, 2022). They were GCN-based models (Kipf and Welling, 2016) on LDAs prediction. For gGATLDA, according to its experimental setup, we correspondingly collected its three benchmark datasets called Dataset1, Dataset2 and Dataset3 to compare the performance. Specifically, Dataset1 contained 3207 LDAs (443 lncRNAs and 608 diseases); Dataset 2 contained 2697 LDAs (240 lncRNAs and 412 diseases) and Dataset3 contained 621 LDAs (285 lncRNAs and 226 diseases). To objectively compare the performance of LDAGRL with the state-of-the-art methods, similarly, we adopt 5-CV to conduct comparison in a targeted manner for false positive rate in identifying novel LDAs by LDAGRL, combining the precision indicator of prediction. As shown in Table 3, gGATLDA show unstable average precision on three kinds of datasets under 5-CV, while LDAGRL exhibits stable average precision under 5-CV and thus lower false positive rate.

**TABLE 3:** The predictive performance comparison of gGATLDA and LDAGRL by 5-CV.

Evaluation indicators	Dataset			
	Dataset1	Datase2	Dataset3	Method
AUC	0.9888	0.9870	0.9442	gGATLDA
AUPR	0.9890	0.9864	0.9493	
Precision	0.7980	0.9098	0.8124	
Accuracy	0.8670	0.9395	0.8455	
Recall	0.9913	0.9759	0.9029	
F1-Score	0.8830	0.9416	0.8541	
AUC	0.9258	0.9167	0.9037	LDAGRL
AUPR	0.9126	0.8892	0.8976	
Precision	0.8508	0.8510	0.8421	
Accuracy	0.8406	0.8390	0.8198	
Recall	0.8260	0.8017	0.8078	
F1-Score	0.8382	0.8256	0.8246	

Though the AUCs and precisions of GCNLDA and GCRFLDA are higher than the corresponding indicator of LDAGRL, it can be observed from Table 4 that three types of methods, including LDAGRL, keep AUC at the same level (>0.90) and the results of experiment manifested that LDAGRL has a trait of low false negative and low false positive.

**TABLE 4:** The predictive performance comparison of three methods by 5-CV.

Evaluation indicators	Method		
	GCNLDA	GCRFLDA	LDAGRL
AUC	0.9589	0.9621	0.9258
Precision	0.8250	0.8278	0.8508

To add an independent baseline approach (i.e., graph embedding algorithm) to compare four types of methods, we adopted the node2vec (Grover and Leskovec, 2016) as the baseline model to obtain node embedding and employed the Random Forest classifier to score the potential LDAs. Since node2vec is a graph embedding algorithm that considers both the Depth First Search (DFS) and the Breadth First Search (BFS) neighborhood. It is consistent with our design idea that exploring the association possibilities between lncRNAs and diseases relies on their bridge nodes or bridge paths.

First, as shown in Table 5, we listed all potential paths and calculated the frequency for lncRNA and disease in BHnet dataset (104,282 edges). Here, the node frequency calculation needs to make sure that each node on the same path appears together (i.e., for L-M-D, we calculate L/D frequency under the condition that L-M-D holds simultaneously, instead of one of L-M, M-D and L-D holds).We can observe that the ‘lncRNA-miRNA-disease’ path can be chosen as a BHnet to conduct baseline (node2vec) due to the highest frequency both for each lncRNA and for each disease.

**TABLE 5:** Bridge paths of between lncRNA and disease in BHnet.

Path	(Node & Path) frequency in BHnet						
	lncRNA (L)	miRNA (M)	Protein (1) (P)	Protein (2) (P)	Drug (Dr)	Disease (D)	Path
lncRNA-miRNA-disease (L-M-D)	477	19				773	31, 0634
lncRNA-protein-disease (L-P-D)	9		19			442	3736
lncRNA-protein-drug-disease (L-P-Dr-D)	8		8		33	574	1, 2818
lncRNA-protein-protein-disease (L-P-P-D)	10		94	359		685	12, 2719
lncRNA-protein-protein-drug-disease (L-P-P-Dr-D)	9		49	131	204	598	56, 6745

Then, we implemented the predictive performance comparison experiment for five methods over node2vec (with default parameters) by 5-CV. As shown in Table 6, five methods can obtain satisfactory AUCs over node2vec, GCRFLDA achieved the best performance on both AUC and Precision. LDAGRL using L-M-D path get the next best performance and its precision is consistently and even higher related to LDAGRL. These results also indicate bridge paths play a key role in LDA prediction.

**TABLE 6:** The predictive performance comparison of five methods over node2vec by 5-CV.

Evaluation indicators	Method					
	GCNLDA	GCRFLDA	gGATLDA		LDAGRL	LDAGRL (L-M-D)
AUC	0.9552	0.9862	0.9640		0.9222	0.9563
Precision	0.9049	0.9442	0.8710		0.8805	0.9162

Besides, GCNLDA, GCRFLDA and gGATLDA all adopted the similarity subnetwork to build heterogeneous bipartite graph or tripartite graph and followed the assumption that the lncRNAs in the same sets are similar. Then they think of LDAs prediction as a recommender systems issue in which they usually view lncRNA as the user and disease as the item. Despite its rationality from a pure computational perspective, it may be controversial and have not stood up to the biological significances. Sometimes, a single nucleotide difference can completely change the nature of a lncRNA. Surely, the coarse-grained feasibility brought about by the way enable LDAs prediction to a certain extent for researchers, but the false positive problems at the same time cannot be neglected. In LDAGRL, we remove the disadvantage of the similarity hypothesis and depend on the regulatory or targeting relationships between lncRNAs and corresponding bridge molecules, obtaining the satisfactory prediction effects with the same level prediction performance and lower false positive rate.

### Comparison experiment results with different classifiers

Network embedding (Yuan et al., 2018) is a crucial method for learning low-dimensional representations of vertexes in network. As described in the method section, the different classifiers may influence LDAGRL prediction performance. Therefore, we implemented the experiment to evaluate the impact of five classifiers.

To evaluate the performance of LDAGRL based on different classifier, we choose XGBoost, Random Forest, Logistic Regression, SVM, and AdaBoost to execute the 5-CV experiment. By validating different classifiers, different values of AUC are obtained through the 5-CV. As seen in Table 7, XGBoost, SVM, Logistic Regression, AdaBoost, and Random Forest are all effective in classification with high AUC values. Moreover, Tree-based classifiers, such as XGBoost and Random Forest, have been demonstrated to be a practical tool in prediction due to their higher operational efficiency and lower over-fitting rate. By looking into the detail of these results, we can observe that, for LDAGRL, compared with the AdaBoost, the XGBoost and the Random Forest achieve higher AUC value. Besides, we can also find that all classifier parameters are default values, and only the bridge feature for nodes is appended to the training process. In the LDAGRL, the result of 5-CV through the XGBoost classifier (with AUC = 0.9258, default parameters) is better than other classifiers. It further verifies the superiority of the SDNE on LDAs prediction.

**TABLE 7:** The AUC results of five classifiers under LDAGRL.

Fold	Classifier				
	XGBoost	Random forest	SVM	AdaBoost	Logistic regression
0	0.9311	0.9112	0.8784	0.8880	0.8930
1	0.9125	0.9102	0.8734	0.8722	0.8802
2	0.9239	0.9308	0.8827	0.8838	0.8899
3	0.9316	0.9290	0.9009	0.9003	0.9031
4	0.9294	0.9324	0.8963	0.8889	0.9021
Average	0.9258	0.9227	0.8863	0.8867	0.8937

### Comparison of different node feature combinations

In the LDAGRL, each node can be represented by its intrinsic attributes and relationship with other nodes. Thus, each node can be represented as a vector (192-dimension) by two kinds of information, including attribute and bridge structure. For attribute information (64-dimensional vectors), the node’s attributes can be the k-mer about sequences of ncRNA and protein, the disease’s semantics, and the drug’s molecular fingerprint. For bridge structure information (128-dimensional vectors), the relationship of each node with others could be abstracted by the network embedding method SDNE.

Here, in comparison with the predictive performance of LDAGRL for different node feature combinations, we mainly divided it into three groups to validate the different performances with Attribute, Bridge, and Attribute + Bridge combinations. It is known that the attribute information is each node’s intrinsic feature, so we design an experiment that can verify the predictive performance of prediction based on attribute information with the previous isolated embedding method.

Furthermore, in the LDAGRL, bridge structure information, as critical relationships among nodes, is vital for LDAs prediction. The main goal we construct LDAGRL is to obtain the network’s relation features. Therefore, it is indispensable to verify the bridge structure’s influence on predictive performance in LDAGRL. After the above two kinds of the feature are verified, considering the complexity of LDAGRL and the character of lncRNAs and diseases, we used the ‘Attribute + Bridge’ combination to evaluate the entire performance, aiming at obtaining optimized features for classifiers and further improve LDAGRL generalization performance.

Among nodes in LDAGRL, bridge structure information is a critical association relationship for LDAs prediction. In other words, the main goal of our constructing LDAGRL is to obtain the relation features, namely, the bridge structure feature.

**FIGURE 4:**
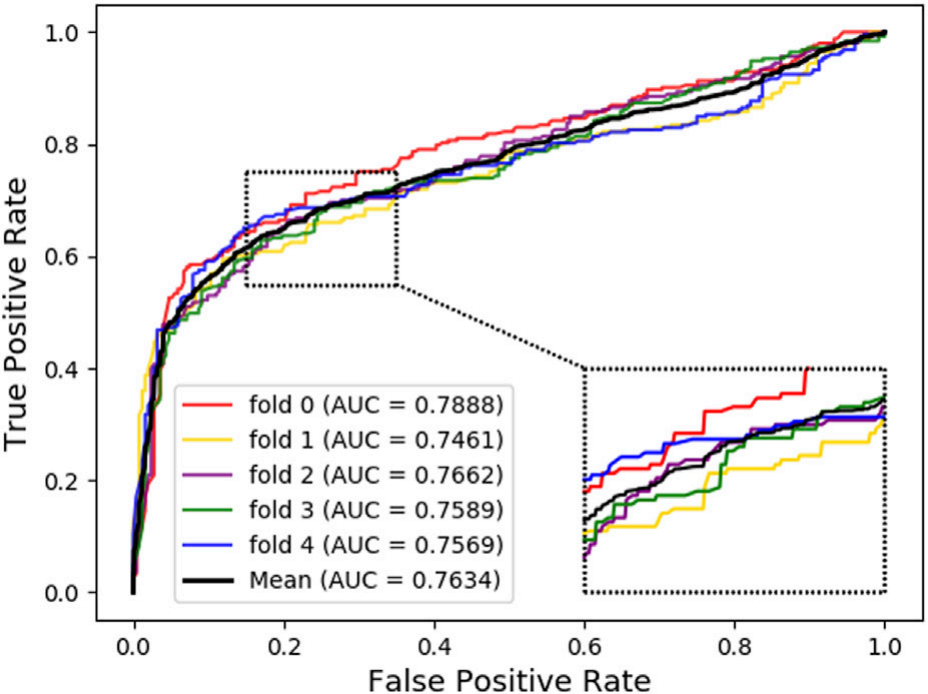
AUC Result based on the ‘Attribute’ feature.

**FIGURE 5:**
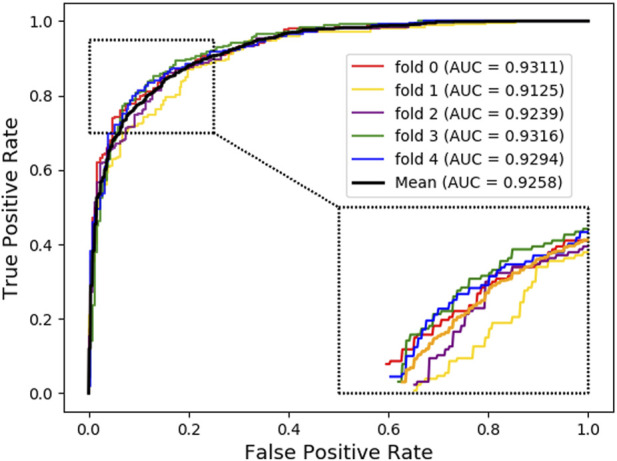
AUC Result based on the ‘Bridge’ feature.

**FIGURE 6:**
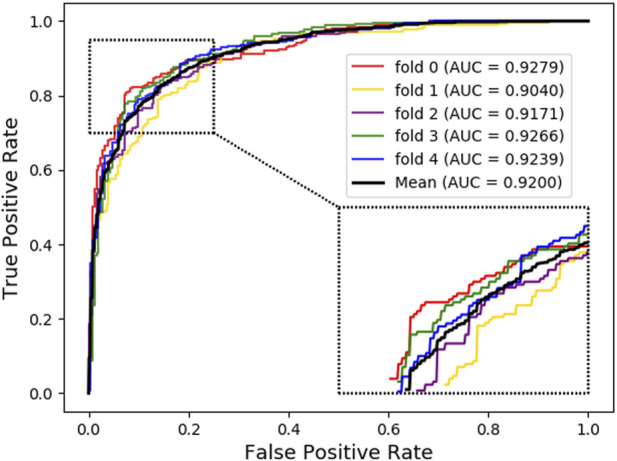
AUC Result based on the ‘Attribute + Bridge’ feature.

As shown below, Figure 4 ~ Figure 6 plot the ROC curves of the three combinations’ results and reports their AUROC values of 5-CV. Figure 4 shows the AUC result of the ‘Attribute’ that 5-CV with pure attribute information as the node’s characteristics. Figure 5 shows the AUC result of ‘Bridge’ that 5-CV with pure bridge structure information as the feature of the node. Figure 6 shows the AUC result of the ‘Attribute + Bridge’ combination based on the feature combined attribute information with the bridge structure.

As seen from Table 8, the ‘Attribute’ combination has the lowest AUC and accuracy rate in LDAGRL. When the feature combination is ‘Attribute + Bridge’, the value of AUC based on SDNE for XGBoost barely change, and the average accuracy rate increase more slowly than the ‘Bridge’ combination. Therefore, the predictive performance of LDAs based on the feature combined attribute information with bridge structure information (i.e., ‘Attribute + Bridge’ combination) is not better than the ‘Bridge’ combination, which dominates the highest TPRs under the same FPRs and has the highest AUC (0.9258). It implies that attribute information has a small impact on predictive performance. Besides, it is worth pointing out that though the augment of features information is relatively large for ‘Attribute + Bridge’, the average accuracy rate of the ‘Bridge’ and ‘Attribute + Bridge’ remain stable. It is because we chose the SDEN to globally represent the bridge structure feature of nodes in the entire network and the flow of information directly or latently with other nodes, thus improving the performance. In addition, the results in Figure 5 show that the ‘Bridge’has superiority in LDAGRL.

**TABLE 8:** Comparison of different feature combinations.

Feature	Acc	Sen	Spec	Prec	MCC
Attribute	0.7290	0.6471	0.8109	0.7746	0.4648
Bridge	0.8406	0.8260	0.8552	0.8508	0.6816
Both	0.8394	0.82.68	0.8521	0.8483	0.6793

### Case study

Endocrine system diseases including type 2 diabetes mellitus, diabetic nephropathy, obesity and osteoporosis are common diseases (Sun et al., 2022). It is evident that the early detection of endocrine system diseases is vital to precise treatment (Hackney and Lane, 2015; Rachdaoui and Sarkar, 2017). Hence, the case study is implemented to identify the possible lncRNAs associated with endocrine system diseases to thus explore the generalization ability of LDAGRL. Specifically, we take LDAGRL to identify novel LDAs and verify the prediction results based on ENCD database (Hao et al., 2023).After scoring those scores for potentially associated lncRNAs with the endocrine system diseases, all predicted disease-related lncRNAs are ranked. Here, we select the top 10 associated lncRNAs which get the highest predicted ranks for endocrine system diseases. Relevant biology literature and databases support predictive results, and the details shown in Table 9. Here, we listed the top 10 predicted lncRNAs and then confirmed them in relevant biology literature or databases, which also indicated the consistency between LDAGRL and biology wet experiments.

**TABLE 9:** Validation of the top 10 lncRNAs for four types of endocrine system diseases.

	Type 2 diabetes mellitus		Diabetic nephropathy	
Rank	lncRNA	PMID	lncRNA	PMID
1	PAX8-AS1	33155514	ARAP1-AS2	31079598
2	LINC01503	32337289	H19	32391614
3	MIR143HG	33274206	NEAT1	30515796
4	GAS5	31849505	CASC2	32016985
5	LINC01173	32337289	ZEB1-AS1	30121551
6	ARAP1	31975379	UCA1	31799676
7	H19	30201684	PVT1	31371698
8	MEG8	32765026	TUG1	31539141
9	PLUTO	28041957	GAS5	31849505
10	XIST	32447981	SNHG17	32627655

	Obesity		Osteoporosis	
Rank	lncRNA	PMID	lncRNA	PMID
1	MALAT1	31659145	CRNDE	30280760
2	H19	ENCD	XIST	33336851
3	PRINS	ENCD	HOTAIR	ENCD
4	XIST	ENCD	KCNQ1OT1	ENCD
5	KCNQ1OT1	ENCD	BCAR4	32572903
6	MAFG	32005828	SNHG14	33928771
7	EDF1	30061575	FTX	32660465
8	HAR1A	ENCD	ENSG00000260802	32742382
9	DMPK	ENCD	LINC01535	33174047
10	MIR31HG	ENCD	DANCR	25660720

## Conclusion

Recently, more and more lncRNAs are identified and their functions associated with multiple diseases have received much attention. We construct a bridge heterogeneous information network based on five nodes and nine kinds of relationships to detect lncRNA-diseases associations. To evaluate the performance of our method, a set of comprehensive experiments are implemented, and the validation results demonstrate the effectiveness of LDAGRL. The prediction performance obtained by LDAGRL could be due to several reasons: first of all, our method integrated associations information of lncRNA, miRNA, diseases, drug, protein, and their associated biomolecules for lncRNA and diseases by constructing a bridge heterogeneous information network, so that the LDAGRL could fully make use of the integrated associated data, which can further enhance its predictive performance as a global network model. Second, each node can be represented as a vector by two kinds of information including node attributes and node bridge structure, which can improve prediction performance. Significantly, the ‘Bridge’can further improve prediction performance and has its superiority.

In conclusion, in this paper, an LDAGRL model is presented, developed, and investigated for the association prediction of the lncRNA-disease pair. The LDAGRL model takes advantage of the bridge heterogeneous information network. The validation results demonstrate that LDAGRL can globally obtain satisfactory performance. In verifying the feasibility and effectiveness of the bridge heterogeneous information network, the proposed LDAGRL and their experiment results show the expected effect on LDAs prediction. Even so, the current version of LDAGRL has limitations. For example, only 1264 known lncRNA-disease associations have been adopted by LDAGRL; the prediction accuracy of LDAGRL will improve if more known LDAs are added.

## Data Availability

The original contributions presented in the study are included in the article/supplementary material, further inquiries can be directed to the corresponding author.
